# Molecular survey and characterization of a novel *Anaplasma* species closely related to *Anaplasma capra* in ticks, northwestern China

**DOI:** 10.1186/s13071-016-1886-6

**Published:** 2016-11-25

**Authors:** Jifei Yang, Zhijie Liu, Qingli Niu, Junlong Liu, Rong Han, Guangyuan Liu, Yaoxu Shi, Jianxun Luo, Hong Yin

**Affiliations:** 1 State Key Laboratory of Veterinary Etiological Biology, Key Laboratory of Veterinary Parasitology of Gansu Province, Lanzhou Veterinary Research Institute, Chinese Academy of Agricultural Science, Xujiaping 1, Lanzhou, Gansu 730046 People’s Republic of China; 2 Jiangsu Co-innovation Center for Prevention and Control of Important Animal Infectious Diseases and Zoonoses, Yangzhou, 225009 People’s Republic of China; 3 China Agricultural Veterinary Biological Sciences and Technology Co., Ltd, Lanzhou, Gansu 730046 People’s Republic of China

**Keywords:** *Anaplasma*, 16S rRNA gene, *gltA* gene, *groEL* gene, *Haemaphysalis qinghaiensis*, *A. capra*-like bacteria, China

## Abstract

**Background:**

*Anaplasma* spp. are tick-transmitted bacteria that infect a wide variety of wild and domestic animals. These pathogens exhibit a high degree of biological diversity, broad geographical distribution, and represent a serious threat to veterinary and public health worldwide.

**Results:**

A novel *Anaplasma* species was identified in *Haemaphysalis qinghaiensis* (Ixodidae) in northwestern China and was molecularly characterized by comparison of 16S rRNA, *gltA*, and *groEL* gene sequences. Of the 414 samples tested, 24 (5.8%) were positive for this *Anaplasma* species. On the basis of the 16S rRNA gene, this organism has been found to be closely related to and exhibit the highest sequence similarity with *A. capra* (99.8–99.9%) that was identified in goats and humans in northern China, but was distinct from other known *Anaplasma* species. Sequence analysis of the *gltA* and *groEL* genes revealed that this *Anaplasma* species was distinct from *A. capra* considering the lower sequence identity (88.6–88.7% for *gltA* and 90.6–91.0% for *groEL*) and a divergent phylogenetic position. Therefore, we described this *Anaplasma* species as *A. capra*-like bacteria.

**Conclusions:**

The present study reports a potential novel *Anaplasma* species closely related to *A. capra* in *H. qinghaiensis* in northwestern China. The zoonotic potential of *A. capra*-like bacteria needs to be further determined.

## Background

Members of the genus *Anaplasma* are gram-negative obligate intracellular bacteria that reside within membrane-enclosed vacuoles in the cytoplasm of blood or endothelial cells [1]. This genus encompasses seven recognized species, which are known to infect mammals and different cell types [1]. *Anaplasma phagocytophilum* infects neutrophils of animals and humans; *Anaplasma marginale*, *Anaplasma centrale* (*A. marginale* subsp. *centrale*), *Anaplasma ovis* and *Anaplasma mesaeterum* infect erythrocytes of ruminants; while *Anaplasma bovis* and *Anaplasma platys* infect bovine monocytes and canine platelets, respectively [2–4]. Recently, a novel *Anaplasma* species designated “*Anaplasma capra*” was identified in goats, ticks, and humans in northern China [5]. In addition to this species, other potential novel *Anaplasma* species or genetic variants have been reported on the basis of phylogenetic analysis of different gene loci in ticks and vertebrate hosts, particularly in wildlife, including *Anaplasma odocoilei* from white-tailed deer and “*Candidatus* Cryptoplasma californiense” from *Ixodes pacificus* in USA, *A. platys*-like strains from cats in Italy, and novel *Anaplasma* sp. from sika deer in Japan and dromedary camels in Saudi Arabia [6–10].

The discovery of novel tick-transmitted *Anaplasma* species indicates that the global burden of anaplasmosis on animal and human health has been underestimated. In general, several emerging tick-borne pathogens were initially identified in ticks or animals, but were identified as human pathogens much later. *Anaplasma phagocytophilum* was first described in 1932 in Scotland as the agent of tick-borne fever in sheep [11]; however, the first case of human granulocytic anaplasmosis (HGA) was recorded in 1994 in the United States [12]. *Anaplasma capra* was initially found in goats, and human infection with this agent was subsequently reported in an active surveillance of patients in a hospital in northern China [5]. The novel identified *Anaplasma* species represent potential candidates for new tick-borne diseases that have enriched our understanding of anaplasmosis. In the present study, a potential novel *Anaplasma* species closely related to *A. capra* was found in *Haemaphysalis qinghaiensis* ticks in a high altitude area in northwestern China.

## Methods

Questing ticks were collected on the vegetation with the flagging method once a month between March and May 2011, in Gannan Tibetan Autonomous Prefecture (33°06"–36°10"N, 100°46"–104°44"E) in Gansu Province. The sampling sites were located in forest and pasturing areas that rely heavily on the farming of sheep, goats, and yaks for milk, and meat for the local economy. The average altitude at the sampling sites is over 3,000 m. Ticks were identified as *Haemaphysalis qinghaiensis* microscopically on the basis of morphological parameters [13]. DNA was extracted from adult *H. qinghaiensis* ticks individually using the Puregene DNA purification kit (Qiagen, Beijing, China) according to the manufacturer’s protocols.

The DNA of 414 tick samples was screened for the presence of the *gltA* gene of *Anaplasma* sp. by nested PCR, with the primers and PCR conditions described in Table 1. The first-round PCR was carried out with previously published primers [5]; the primers for the second-round amplification and the primers targeting 16S rRNA and *groEL* genes were designed based on the corresponding sequences of *A. capra* HLJ-14 using Primer Premier 5.0 software (PREMIER Biosoft International, 3786 Corina way, Palo Alto, CA, USA) [5]. In order to further identifying the agent, the partial 16S rRNA and the *groEL* gene fragments were amplified from samples positive for *gltA* gene of the *Anaplasma* sp. (Table 1). PCR reactions were performed in an automatic thermocycler (Bio-Rad, Hercules, USA). Genomic DNA extracted from infected ticks that had been verified by sequencing was used as the positive control, and sterile water was used as the negative control. PCR products were visualized by UV transillumination in a 1.0% agarose gel following electrophoresis and staining with ethidium bromide.

**Table 1:** Primers and PCR amplification conditions

Target gene	Primer name	Primer sequence (5′–3′)	Annealing temperature (°C)	Amplicon size (bp)	Reference
*gltA*	Outer-f	GCGATTTTAGAGTGYGGAGATTG	55	1,031	[5]
	Outer-r	TACAATACCGGAGTAAAAGTCAA			
	Inner-f	TCATCTCCTGTTGCACGGTGCCC	60	594	This study
	Inner-r	CTCTGAATGAACATGCCCACCCT			
16S rRNA	Forward	GCAAGTCGAACGGACCAAATCTGT	58	1,261	This study
	Reverse	CCACGATTACTAGCGATTCCGACTTC			
*groEL*	Forward	TGAAGAGCATCAAACCCGAAG	55	874	This study
	Reverse	CTGCTCGTGATGCTATCGG			

The PCR products of the *gltA* (594 bp), 16S rRNA (1,261 bp) and *groEL* (874 bp) genes were purified (TaKaRa Agarose Gel DNA purification Kit Ver. 2.0, Dalian, China), cloned (pGEM-T Easy vector, Promega, Madison, WI, USA) and subjected to sequencing using BigDye Terminator Mix (Sangon, Shanghai, China). The GenBank accession numbers of *Anaplasma* sp. detected in *H. qinghaiensis* ticks in this study are as follows (not including identical sequences): KX673824 and KX673825 (16S rRNA), KX685885 and KX685886 (*gltA*) and KX685887 and KX685888 (*groEL*). Sequences were compared with the published sequences in GenBank by a BLASTn search and analyzed with the Clustal W method in the MegAlign software (DNAStar, Madison, WI, USA). Phylogenetic analysis was conducted based on the sequence distance method using the neighbor-joining (NJ) algorithm with the Kimura two-parameter model of the Mega 4.0 Software [14]. The results were analyzed using a Chi-square test in Predictive for Analytics Software Statistics 18 (PASW, SPSS Inc., Chicago, IL, USA). *P*-values of 0.05 or less were considered statistically significant.

## Results and discussion

In this study, DNA of an *Anaplasma* species was detected in *H. qinghaiensis* from Gannan Tibetan Autonomous Prefecture in Gansu Province, northwestern China. *Haemaphysalis qinghaiensis* is a distinctive tick species that is common in high altitude areas in northwestern China, and preferentially infests domestic animals such as sheep, goats, cattle and yaks [13, 15, 16]. Out of the 414 *H. qinghaiensis* ticks sampled, 24 (5.8%) were positive for the *Anaplasma* sp. The infection rates of the *Anaplasma* sp. were comparable in female (5.7%, 13/230) and male (6.0%, 11/184) ticks (*χ* ^2^ = 0.02, *P* > 0.05).

**Fig. 1:**
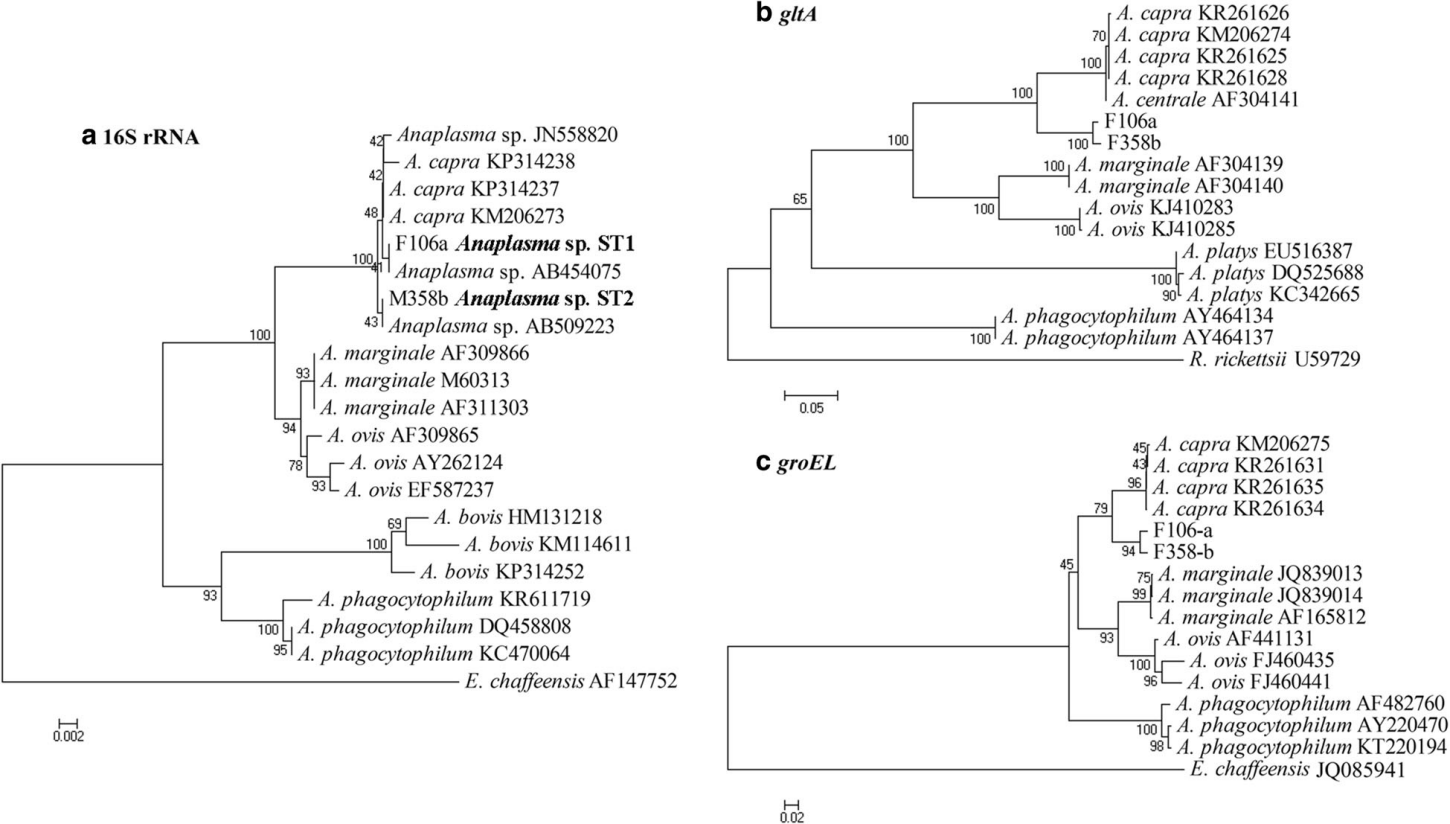
Phylogenetic analysis of the *Anaplasma* species identified in this study based on the 16S rRNA (**a**), *gltA* (**b**) and *groEL* (**c**) genes. *Ehrlichia chaffeensis* and *Rickettsia rickettsii* were used as outgroups

The *Anaplasma* sp. identified in *H. qinghaiensis* ticks was further characterized based on 16S rRNA, *gltA*, and *groEL* genes. Sequence analysis showed that the 16S rRNA gene sequences (1,261 bp) were classified into two sequence types (ST), with 99.9% similarity, representing two different *Anaplasma* strains. *Anaplasma* sp. ST1 and ST2 (GenBank accession nos. KX673824 and KX673825) were 100% identical to the strain NS104 and Kamoshika17 of unclassified *Anaplasma* species (GenBank accession nos. AB454075 and AB509223) that have been detected in deer and *Capricornis crispus*, respectively, in Japan. Furthermore, the 16S rRNA gene sequences of these isolates were 99.8–99.9% identical (differed by one or two nucleotides) to strain HLJ-14 of the emerging zoonotic *A. capra* (GenBank accession no. KM206273) that was reported in goats and humans in China [5]. Phylogenetic analyses based on 16S rRNA gene sequences revealed that the *Anaplasma* sp. ST1 and ST2 were in the same clade as members of *Anaplasma* (Fig. 1). These isolates were closely related to *A. capra*, but distinct from other known *Anaplasma* species (Fig. 1).

Further analyses of *gltA* and *groEL* gene sequences showed 98.8% and 98.0% similarity between the *Anaplasma* sp. ST1 and ST2, respectively. The *gltA* sequences of *Anaplasma* sp. ST1 and ST2 (GenBank accession nos. KX685885 and KX685886) were 88.7% and 88.6% identical to *A. capra* (GenBank accession no. KM206274). The *groEL* sequences of *Anaplasma* sp. ST1 and ST2 (GenBank accession nos. KX685887 and KX685888) were 91.0% and 90.6% identical to *A. capra* (GenBank accession no. KM206275). Phylogenetic analysis based on *gltA* and *groEL* sequences showed that the *Anaplasma* sp. ST1 and ST2 clustered independently from *A. capra* and other *Anaplasma* species with high bootstrap values (Fig. 1), indicating potential novelty of the studied *Anaplasma* sp.

Ticks are important vectors of various pathogens that affecting domestic and wild animals as well as humans worldwide [17]. With the development of molecular tools, emerging tick-borne pathogens and the increasing number of tick-associated disease cases were identified in tropical and sub-tropical areas [18–20], suggesting that there are still new tick-borne pathogens to be discovered. In this study, an *Anaplasma* species was identified in *H. qinghaiensis* ticks in northwestern China. The *Anaplasma* sp. ST1 and ST2 were closely related to *A. capra* on the basis of 16S rRNA gene (similarity of 99.8–99.9%). However, sequence and phylogenetic analyses based on the *gltA* and *groEL* genes indicated that the *Anaplasma* sp. (ST1 and ST2) differed from *A. capra* considering the lower sequence identity and divergent phylogenetic position. According to the results presented here, we described the *Anaplasma* species identified from *H. qinghaiensis* ticks as *A. capra*-like bacteria.

The members of the genus *Anaplasma* are now recognized to be important human and animal pathogens [21]. To date, two *Anaplasma* species have been identified as pathogens of human anaplasmosis [5, 12]. *Anaplasma phagocytophilum* has been well studied and viewed as zoonotic pathogen for years already. *Anaplasma capra* was initially found in asymptomatic goats, and case of human infection were confirmed in 2015 in Heilongjiang province, northern China [5]. The agent was also detected in *Ixodes persulcatus* in Heilongjiang province and *Haemaphysalis longicornis* ticks in Shandong Province [22]. The illness caused by *A. capra* are different from *A. phagocytophilum* infection [5]. In this study, *A. capra*-like bacteria were identified in *H. qinghaiensis* ticks. However, it is still not clear whether this *Anaplasma* species is pathogenic to humans and animals.

The reservoir hosts of *Anaplasma* play a critical role in the maintenance of the pathogens in nature. As already mentioned, sequences that were identical to the *A. capra*-like bacteria have been detected in deer (*Anaplasma* sp. NS104, GenBank accession no. AB454075) and in free-living *Capricornis crispus* (*Anaplasma* sp. Kamoshika17, GenBank accession no. AB509223) in Japan [23]. Natural infections with these isolates in deer and *Capricornis crispus* suggested that the *A. capra*-like bacteria may be maintained in nature through enzootic cycles between ticks and wild animals. Several domestic and wild animal species as hosts of *H. qinghaiensis* ticks indicated that those animals could be reservoir hosts for the *A. capra*-like bacteria in study sites. The isolation of the organism from ticks and infected animals may help further elucidate the pathogenesis and characteristics of this *Anaplasma* species.

## Conclusions

The present study reported a potential novel *Anaplasma* species closely related to *A. capra* in ticks in China. Twenty-four (5.8%) of the 414 *H. qinghaiensis* ticks sampled were positive for the *Anaplasma* species. On the basis of the sequence and phylogenetic data, we described the *Anaplasma* species as *A. capra*-like bacteria.

## Abbreviations

*gltA*: Citrate synthase
*groEL*: Heat-shock operon
ST: Sequence type
